# Interactions Between Gut Microbiota, Host, and Herbal Medicines: A Review of New Insights Into the Pathogenesis and Treatment of Type 2 Diabetes

**DOI:** 10.3389/fcimb.2020.00360

**Published:** 2020-07-17

**Authors:** Yujiao Zheng, Xiaowen Gou, Lili Zhang, Hanjia Gao, Yu Wei, Xiaotong Yu, Bing Pang, Jiaxing Tian, Xiaolin Tong, Min Li

**Affiliations:** ^1^Department of Endocrinology, Guang'anmen Hospital, China Academy of Chinese Medical Sciences, Beijing, China; ^2^Graduate School, Beijing University of Chinese Medicine, Beijing, China; ^3^Molecular Biology Laboratory, Guang'anmen Hospital, Chinese Academy of Traditional Chinese Medicine, Beijing, China

**Keywords:** herbal medicines, type 2 diabetes mellitus, gut microbiota, bacterial metabolites, therapeutic mechanisms

## Abstract

Herbal medicines (HMs) are a major subset of complementary and alternative medicine. They have been employed for the efficient clinical management of type 2 diabetes mellitus (T2DM) for centuries. However, the related underlying mechanisms still remain to be elucidated. It has been found out that microbiota is implicated in the pathogenesis and treatment of T2DM. An interplay between gut microbiota and host occurs mainly at the gastrointestinal mucosal barrier. The host movements influence the composition and abundance of gut microbiota, whereas gut microbiota in turn modulate the metabolic and immunological activities of the host. Intestinal dysbiosis, endotoxin-induced metabolic inflammation, immune response disorder, bacterial components and metabolites, and decreased production of short-chain fatty acids are considered significant pathogenic mechanisms underlying T2DM. The interaction between gut microbiota and HMs during T2DM treatment has been investigated in human, animal, and *in vitro* studies. HMs regulate the composition of beneficial and harmful bacteria and decrease the inflammation caused by gut microbiota. Furthermore, the metabolism of gut microbiota modulates HM biotransformation. In this review, we have summarized such research findings, with the aim to improve our understanding of the pathogenesis and potential therapeutic mechanisms of HMs in T2DM and to provide new insights into specific targeted HM-based therapies and drug discovery.

## Introduction

Diabetes mellitus (DM) is a widely prevalent chronic disease associated with significant healthcare problems worldwide. According to the International Diabetes Federation (IDF), ~425 million adults suffered from DM in 2017, and the number is expected to increase to 629 million by 2045 (Cho et al., 2018). The prevalence of DM varies between developing and developed countries and is estimated to increase in these countries by 69 and 20% from 2010 to 2030, respectively (Shaw et al., 2010). Long-term DM can result in multiple complications and comorbidities, affecting the eyes, kidneys, cardiovascular system, and nervous system (Inzucchi et al., 2012). The mortality rates of adult patients with DM are reportedly two to four times higher than in adults without diabetes (Gerrits et al., 2015). Type 2 diabetes mellitus (T2DM) is one of the main types of DM. It is characterized by hyperglycemia resulting from progressive β-cell dysfunction in the presence of insulin resistance (American Diabetes Association, 2019). Furthermore, T2DM accounts for more than 90% of all diabetic patients, leading to a public pandemic (Chatterjee et al., 2017). The prevalence of T2DM has increased markedly over the years, causing a significant global burden of mortality and disability (Zheng et al., 2017). Obesity is the most prominent risk factor for T2DM. Other risk factors include a low-fiber diet and an unhealthy lifestyle, including inactivity, smoking, and alcohol consumption (Wu et al., 2014; Kautzky-Willer et al., 2016). There are various therapeutic approaches to T2DM, such as lifestyle management (Schellenberg et al., 2013) (self-management education and support, nutrition therapy, physical activity, dietary planning, and psychosocial care), oral medications (Qaseem et al., 2017) (metformin, sulfonylureas, thiazolidinediones, α-glucoside inhibitors, dipeptidyl peptidase-4 inhibitors, and sodium-dependent glucose transporter 2), injectable medications (Tran et al., 2015) (insulin and glucagon-like peptide-1), surgery (Maleckas et al., 2015), and complementary and alternative medications (Nahas and Moher, 2009).

With the rising importance of complementary and alternative medicine according to the recommendations of the World Health Organization (WHO) (Zhang Q. et al., 2019), the application and research of alternative medications for the treatment of T2DM have markedly increased in recent years (Al-Eidi et al., 2016; Pang et al., 2019). As one of the paramount types of alternative medicine, herbal medicine (HM) has thousand years of history and includes systematic medical theories based on long periods of phenotype-based and personalized clinical trials (Li and Weng, 2017). The WHO estimated that 70–80% of populations living in developing countries considered HM a primary healthcare approach, while the studies of HM are still at the preliminary stage and need further research on the efficacy mechanisms (Ekor, 2014). During the past two decades, HM has played an active role in the treatment of DM and has proved to be valuable for the prevention of disease progression in both European and Asian countries (Banjari et al., 2019). According to the world ethnobotanical information, ~800 herbs have been applied for the control of DM (Alarcon-Aguilara et al., 1998). A large amount of clinical trials and animal tests have demonstrated the effect of various forms of HM, such as the use of single herbs and their extracts (Pang et al., 2015; Mirfeizi et al., 2016), herbal medicine decoctions (Zhang et al., 2011; Ryuk et al., 2017), and Chinese patent medicines (Pang et al., 2017; Chen et al., 2019). Besides establishing the efficacy of HMs against T2DM, studies have also aimed to identify their therapeutic mechanisms using modern science and technology; however, these mechanisms remain to be elucidated.

The gut represents the largest digestive and immune organ of the human body, which harbors trillions of microbes. The microbes inhabiting the gut, also known as gut microbiota, compose a complex ecological community and greatly impact the host health (Lozupone et al., 2012). Nowadays, an increasing number of researchers have focused on the role of gut microbiota in disease and drug treatment, including the interaction of gut microbiota, T2DM, and HM. New perspectives based on gut microbiota have provided interesting insights into the mechanism of the action of HMs in T2DM treatment.

Here, we aim to provide an overview of the relationship among gut microbiota, host, and T2DM from a pathological perspective, including changes of gut microbiota and how they interact with the host in T2DM. Next, we describe the interaction of gut microbiota, host, and HMs in T2DM treatment, which facilitates the understanding of the potential therapeutic mechanisms of HMs. Finally, we summarize and discuss the HM therapeutic strategy based on gut microbiota and present our perspectives.

## Host, Gut Microbiota, and T2DM: Pathogenesis

### Interplay Between Gut Microbiota and Host

The human intestine is a complex ecosystem. The microbiota and host share an extensive platform for intercellular signaling and defense against potential pathogens (Sekirov et al., 2010). In healthy humans, ~3.8 × 10^13^ bacteria colonize the intestine, which together code for over three million genes (Qin et al., 2010). Classifying bacteria by the phylogenetic diversity of variable nucleotide sequences of small subunit ribosomal RNA operons or 16S rRNA genes allows the analysis of the huge microbial community. A diversity of organismal assemblages can yield a core unit at the functional level, and deviations from this core are associated with different physiological states (Turnbaugh et al., 2009).

Microbiome analysis has revealed that disease progression is associated with changes in the fecal microbiome. Research on animal models has indicated that different responses based on host genotypes may contribute to the development of metabolic disorder phenotypes linked with gut microbiota alterations (Miranda-Ribera et al., 2019; Wang J. H. et al., 2019). In healthy individuals, most intestinal bacteria can be classified under five phyla, *Firmicutes, Bacteroidetes, Actinobacteria, Proteobacteria*, and *Verrucomicrobia* (Tremaroli and Backhed, 2012). All these microbes express genes for the production short-chain fatty acids (SCFAs) (Krautkramer et al., 2016), ligands for G-protein-coupled receptors (GPCRs) (Cohen et al., 2017), neurotransmitters (Asano et al., 2012), and other metabolites. In turn, the metabolites genetically and epigenetically influence the host responses (Sharkey et al., 2018). As for the hosts, intrinsic and extrinsic factors both influence the gut bacterial composition. Through delivery at birth, infant feeding, genetics, infections, medications, and diet, the host internal environment changes frequently, leading to congruent alterations of the gut microbiota (Wen and Duffy, 2017). Basically, the host vital movements influence the gut microbiota abundance, whereas the gut microbiota control the metabolic physiological state and immunological functions of the host through a series of gene-regulated mechanisms.

Most of the interactions between the host and gut microbiota occur at the gastrointestinal barrier, which consists of two parts: bacterial and mucosal barriers. Both are crucial for the prevention of passage of commensal bacteria, pathogens, and food antigens from the lumen into the gut tissue and host systemic circulation (Sorini et al., 2019). The bacterial barrier is the first line of defense against luminal content penetration and performs numerous biological functions. The gut microbiota differs along the longitudinal axis of the gut. Thus, various bacteria colonize different places in the intestine. Due to the radial oxygen gradient, microbes residing on the colonic mucosa harbor higher oxygen tolerance and catalase expression compared to luminal or stool-associated bacteria (Albenberg et al., 2014). As for the mucosal barrier, the intestinal epithelium contains a large surface that is lined by a monolayer of intestinal epithelial cells (IECs) (Wu et al., 2019). They create mucosal barriers including both physical and chemical barriers for the maintenance of a symbiotic relationship between the gut microbiota and host (Okumura and Takeda, 2017). Once the intestinal barrier is disrupted, some of the intestinal physiological functions will be impaired, which may cause circular responses of intestinal dysbiosis, inflammation, and enzymatic machinery and immune response disorders.

### How the Gut Microbiota Affect T2DM

#### Intestinal Dysbiosis

Dysbiosis is characterized by a loss of beneficial microorganisms, an expansion of potentially harmful microbes and/or a loss of overall microbial diversity (Olesen and Alm, 2016). Obesity and polymetabolic disorders lead to an imbalance of gut microbiota, which is considered a characteristic of T2DM. Intestinal dysbiosis leads to the translocation of bacterial metabolites, such as trimethylamine (TMA), mediators of metabolic dysregulation, and pathogen-associated molecular patterns (PAMPs) (Tilg et al., 2020). *Bacteroidetes* and *Firmicutes* are the two dominant bacterial phyla in T2DM patients gut microbiota. Interestingly, the ratio of *Bacteroidetes* to *Firmicutes* has been positively and significantly correlated with plasma glucose concentrations in many previous studies (Turnbaugh et al., 2009; Larsen et al., 2010; Schwiertz et al., 2010). The *Bacteroidetes* content rises with weight loss and low-calorie diet, which is beneficial to the recovery from T2DM (Ley et al., 2006). In T2DM patients, the abundance of lipopolysaccharide-producing *Escherichia coli* (phylum *Proteobacteria*) increases. They contribute to enhanced systemic inflammation of the intestine (Qin et al., 2012). Contrarily, *Akkermansia muciniphila* and *Faecalibacterium prausnitzii* are highly abundant human gut microbes in healthy individuals, and their reduced levels are associated with inflammation and alterations of metabolic processes involved in the development of T2DM (Verhoog et al., 2019). The significance of *Akkermansia muciniphila* for the maintenance of the gastrointestinal tract integrity has recently been identified. Its metabolites affect a number of transcription factors and genes involved in cellular lipid metabolism, which is crucial for the precession of metabolic syndrome and T2DM. *Akkermansia muciniphila* and its metabolite propionate modulate the expression of Fiaf, GPR43, histone deacetylases (HDACs), and peroxisome proliferator-activated receptor gamma (PPAR gamma), and they play an important role in the regulation of transcription factor function, cell cycle, lipolysis, and satiety (Lukovac et al., 2014). Intestinal dysbiosis alters the microbiome to upregulate the membrane transport of sugars and transport of branched-chain amino acids and to downregulate butyrate biosynthesis (Luca et al., 2019). The enriched microbial genes mapped to oxidative stress signaling suggest a direct link between the altered microbiota composition and inflammatory state in patients with T2DM (Tilg et al., 2020). The dysbiosis-provoked rupture of the gut barrier leads to local and systemic inflammation, which is relevant to the development of T2DM (Belizario et al., 2018).

#### Endotoxin-Promoted Metabolic Inflammation

T2DM is, to a certain degree, an inflammatory disease, and several inflammatory molecules are indicative of the development of T2DM (Zhou W. Y. et al., 2019). Microbiota modulate the expression of PPARs in intestinal epithelial and immune modulatory cells and alter the host inflammatory responses (Hasan et al., 2019). Meanwhile, homeostatic imbalance or disruption facilitates the translocation of endotoxins like lipopolysaccharide (LPS) into the circulation, which results in enhanced systemic and intestinal inflammation and gastrointestinal wall permeability. In diabetes, the gut microbiota contribute to the pathophysiological regulation of endotoxemia and increase the intestinal permeability due to malfunction of tight junction proteins, such as occuludin and ZO-1 (Cani et al., 2007b, 2008). This increases the plasma levels of LPS, which causes low-grade inflammation in the circulation and eventually, insulin resistance (IR) (Cani et al., 2007a). As a coreceptor for the monocyte differentiation antigen CD14^+^, TLR4 mediates various LPS-induced inflammatory cascades and the development of the innate immune response, which consists of recognition receptors (PRRs), antimicrobial peptides, and secreted IgA (Creely et al., 2007; Jialal and Rajamani, 2014). Chronic low-grade inflammation ensues with the activation of proinflammatory pathways, contributing to obesity, IR, pancreatic β-cell decline, and eventually T2DM (Lew et al., 2019). LPS is one of the PAMPs, which are recognized by the PRRs, including the Toll-like receptors (TLRs) and Nod-like receptors (NLRs). The interaction between PRRs and LPS induces cytokine and interferon production. In turn, they trigger proinflammatory signaling cascades in human peripheral tissues (Zhao C. et al., 2019). Inflammation promotes an oxidative state, which enhances the enrichment of aerotolerant phyla. It also increases the production of terminal electron acceptors for facultative anaerobes. This means that the inflammatory state contributes to the severity of the intestinal dysbiosis, which promotes the destruction of the bacterial barrier (Winter et al., 2013).

#### Reduced Short-Chain Fatty Acid Production

SCFAs are byproducts of anaerobic microbial fermentation of undigested food in the large intestine (Wisniewski et al., 2019). They can modulate the host energy homeostasis through interactions between chemosensory enteroendocrine cells, which belong to epithelial cells and can supply energy themselves (Kuwahara, 2014). There is evidence that SCFAs increase the pancreatic β-cell mass and insulin secretion, reduce glucagon secretion, and regulate glucose metabolism (Mandaliya and Seshadri, 2019b). Intestinal dysbiosis may change the ratio of anaerobic and aerobic bacteria, which leads directly to SCFA reduction. SCFAs, including propionate, butyrate, and acetate, can trigger the local release of peptide YY (PYY) and GLP1. SCFA receptors are highly expressed on GLP1-producing L cells in the distal ileum and colon (Mandaliya and Seshadri, 2019a). Propionate is a substrate for gluconeogenesis that protects the host from diet-induced obesity and glucose intolerance (Ohira et al., 2017). Butyrate, a SCFA fermented by microbiota, is an energy substrate for colonocytes that decreases the permeability of the intestinal barrier by promoting GLP-2 release and increasing mucus secretion (Sonnenburg and Backhed, 2016). Butyrate may suppress the abnormally increased proliferation of colonic epithelial cells in diabetes by targeting HMGB1 (Wang S. Y. et al., 2019). Furthermore, as a histone deacetylase (HDAC) inhibitor, butyrate can promote β-cell differention, proliferation, function and improve insulin, which has a close link with diabetes (Khan and Jena, 2015). Researchers also demonstrated that butyrate and propionate activate intestinal gluconeogenesis (IGN) via cAMP-dependent mechanism and a gut-brain neural circuit involving the fatty acid receptor FFARS, which had a metabolic benefits on glucose control in mice (De Vadder et al., 2016). As for acetate, it's found that increased production of this SCFA promotes increased glucose-stimulated insulin secretion through activation of the parasympathetic nervous system (Perry et al., 2016).

**Figure 1:**
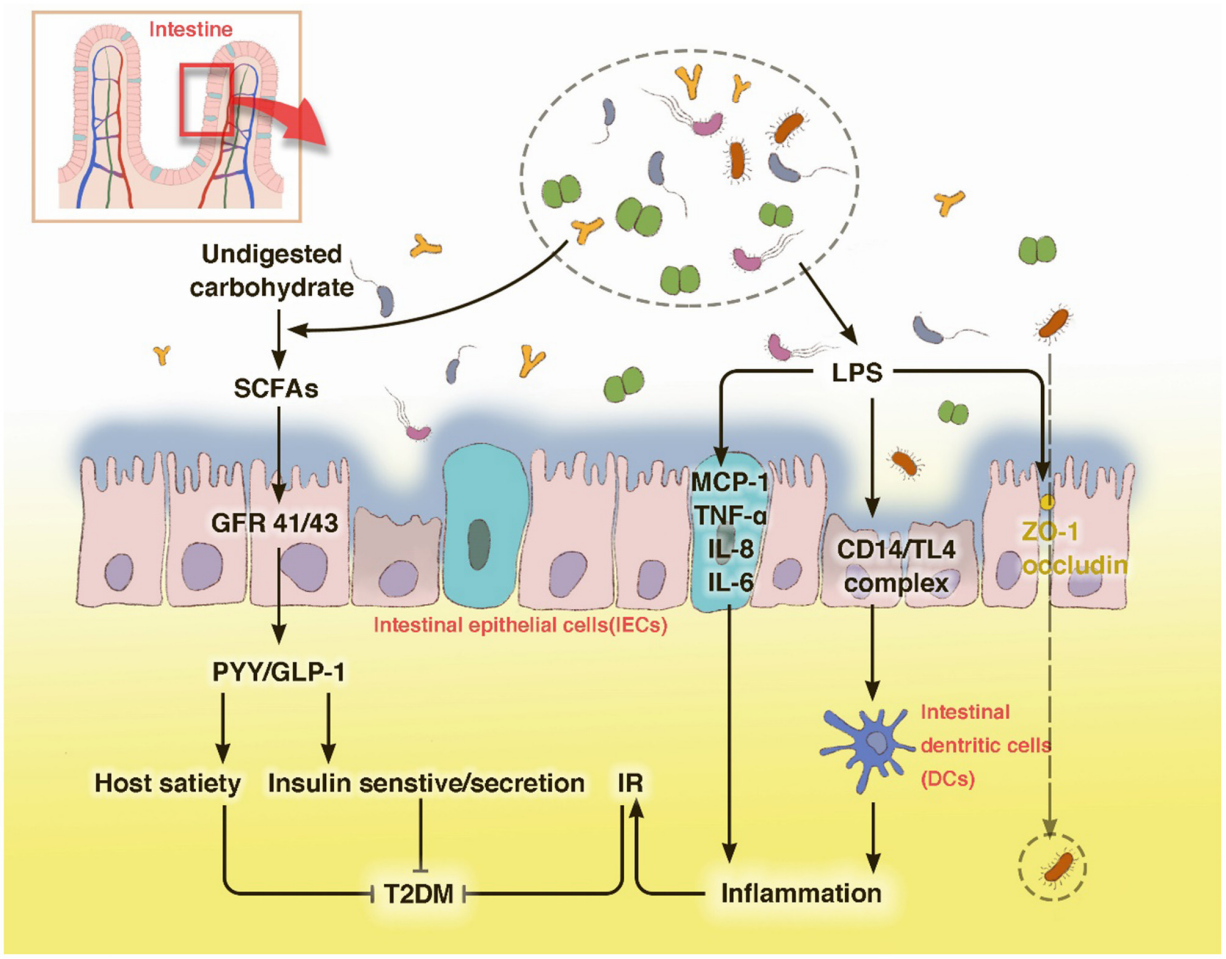
T2DM modulates the abundance of specific gut bacteria to decrease mucus-producing species and several short chain fatty acid (SCFA)-producing bacteria. These bacteria then produce LPS, which can destroy the barrier function of the gut and leak out to the internal environment through occludin and ZO-1, leading to an inflammatory status. The low circulating levels of SCFAs can foster host satiety disorder and a decrease in insulin sensitivity or secretion through GPRs. The disturbance of carbohydrate metabolism may cause an intestinal barrier disruption, which leads to a severer intestinal dysbiosis. With LPS stimulation, IECs secrete the pro-inflammatory cytokines MCP-1, TNF-α, IL-18, and IL-6. Innate immune cells, such as macrophages and DCs, use pattern recognition receptors to attach to pathogens or toxins, such as LPS, which leads to an activation of the inflammatory cascade.

In summary, due to the intestinal dysbiosis, the endotoxin-mediated promotion of metabolic inflammation and reduction of SCFA levels are the two main mechanisms underlying the interplay between gut microbiota and host in T2DM (Figure 1).

#### Metabolic Dysfunction Induced by Bacterial Components and Metabolites

**Figure 2:**
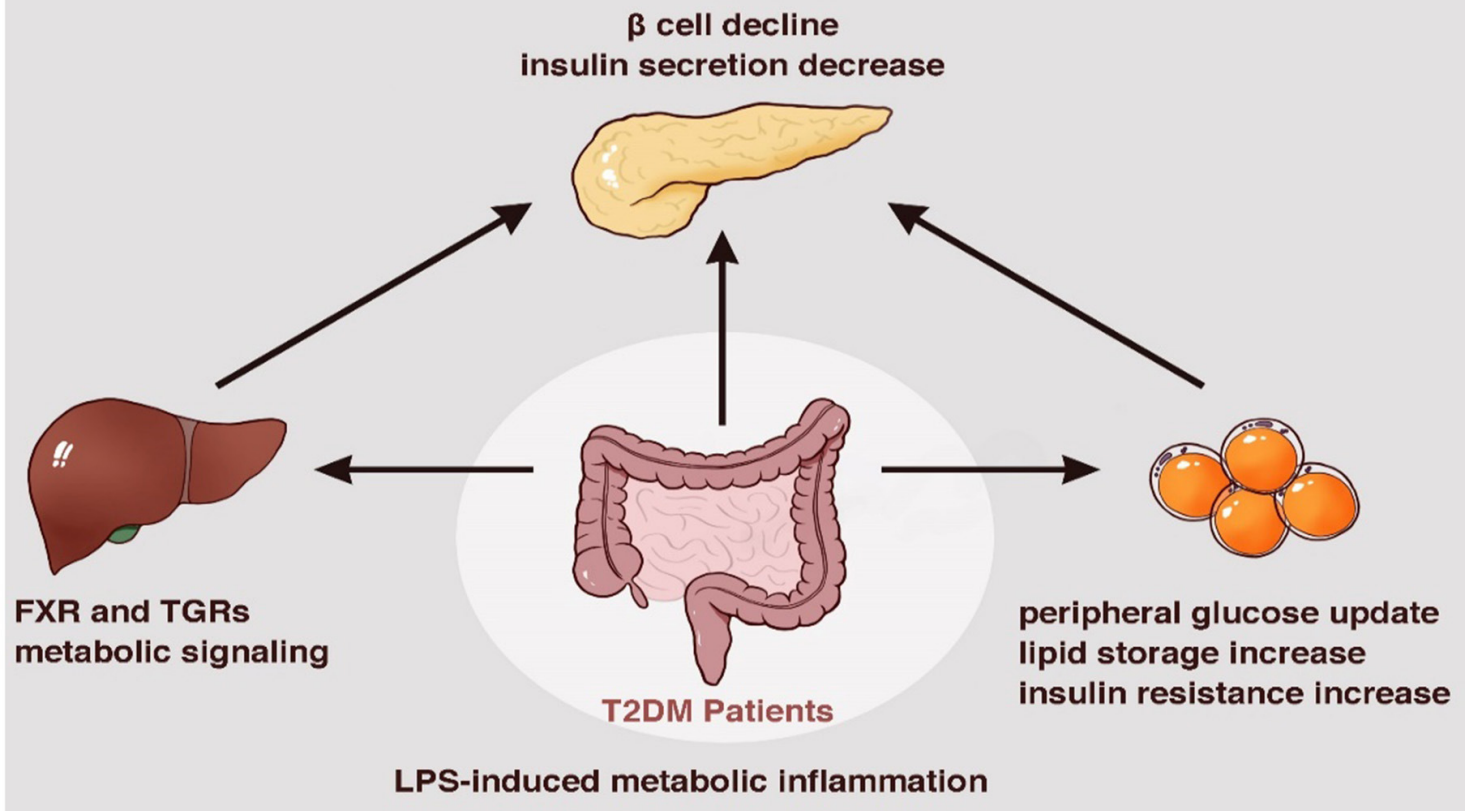
Metabolic dysfunction induced by bacterial components and metabolites a series of enzymatic machinery reactions, leading to insulin resistance.

During food digestion, xenobiotic metabolism and vital movements of the host activate a series of events via enzymatic pathways to function in combination with the gut (Tremaroli and Backhed, 2012). Bacterial components contribute to the production of many bioactive molecule types like bile acids and adipokines, which are essential for the interconnected pathways of glycolysis, tricarboxylic acid/Krebs cycle, oxidative phosphorylation (OXPHOS), and amino acid and fatty acid metabolism (Belizario et al., 2018). Bile acids are molecules generated from cholesterol by microbiota of the lower small intestine and colon. They reportedly inhibit diet-induced obesity and prevent the development of IR by activating the bile acid receptor (farnesoid × receptor, FXR) and membrane G protein coupled receptor TGR5, indicating their effects on energy homeostasis (Gastaldelli et al., 2010; Gerard and Vidal, 2019). The ability to metabolize the naturally occurring FXR antagonist tauro-b-muricholic acid is an essential step toward impaired tolerance to glucose and insulin (Lazar et al., 2019). Adipose tissue is commonly recognized as an active organ presenting key metabolic and endocrine functions by secreting bioactive peptides and proteins, referred to as adipokines, which play a critical role in host metabolism. With the impact of gut microbiota it releases inflammatory adipokines, such as fatty acid binding protein 4 (FABP-4), acylation-stimulating protein (ASP), retinol-binding protein 4 (RBP4), lipocalin-2 (LCN2), and chemerin, etc., which are associated with increased inflammation, obesity, insulin resistance, and eventually T2DM (Lee et al., 2019) (Figure 2).

## Host-Gut Microbiota-Herbal Medicine Interaction During T2DM Treatment

### HMs Regulate the Composition of Beneficial and Maleficent Bacteria

A significant difference between the gut microbiota composition of T2DM patients and healthy individuals was discovered as early as in 2010. The ratios of *Bacteroidetes* to *Firmicutes* and the *Bacteroides-Prevotella* group to *C. coccoides-E. rectale* group were closely related to blood glucose concentration; furthermore, members of *Betaproteobacteria* were highly enriched in T2DM patients (Larsen et al., 2010). With the increasing evidence from research, the imbalance of beneficial and maleficent bacteria is considered important for the pathogenesis of T2DM. SCFAs, including butyrate, propionate, and acetate, are associated with attenuated obesity and T2DM (Ju et al., 2019). The SCFA butyrate is especially beneficial for the improvement of T2DM by increasing insulin sensitivity and enhancing mitochondrial function (Gao et al., 2009). The increase in the abundance of SCFA-producing, and especially, butyrate-producing bacteria and the decline of the abundance of opportunistic pathogens are crucial mechanisms underlying T2DM treatment (Zhang B. et al., 2019).

Rhubarb is a perennial herb used for the therapy of inflammatory diseases including acute pancreatitis and gastroenteritis and of diabetes and its complications in combination with other herbs (Li et al., 2004; Zhou et al., 2016; Cao et al., 2017). Anthraquinone, which contains free anthraquinones and glycosides, is one of its major components (Arvindekar et al., 2015). A recent animal study found that the therapeutic mechanism of a purified anthraquinone-glycoside preparation from rhubarb (RAGP) for T2DM involves an improvement of gut dysbiosis with an enrichment of probiotic *Lactobacillus* and short-chain fatty acid-producing bacteria and decreased abundance of the *Lachnospiraceae NK4A136* group and LPS-producing bacteria *Desulfovibrio* (Cui et al., 2019).

Water extract of *Caulis Spatholobi* (WECS) effectively maintained blood glucose homeostasis and reduced insulin resistance in a study using a diet-introduced obesity (DIO) mouse model. It improved not only laboratory indicators related to diabetes but also microbiota dysbiosis, mainly by increasing the abundance of anti-obesity and anti-diabetes-related bacterial genera, including *Parabacteroides, Bacteroidetes, Anaerotruncus*, and *Bifidobacterium* (Zhang C. et al., 2019).

Xiexin Tang (XXT) is a Chinese herbal formula commonly applied for the treatment of diabetes. In an *in vivo* study, XXT notably shifted the gut microbiota of T2DM rats. It increased the abundance of SCFA-producing and anti-inflammatory bacteria, such as *Adlercreutzia, Alloprevotella, Barnesiella*, [*Eubacterium*] *Ventriosum* group, *Blautia, Lachnospiraceae UCG-001, Papillibacter*, and *Prevotellaceae NK3B31* group to different degrees. The changes of gut microbiota composition were consistent with the amelioration of T2DM rat hyperglycemia, lipid metabolism disorder, and inflammatory activities (Wei et al., 2018).

Huang-Lian-Jie-Du decoction (HLJDD), a famous Chinese herbal formula originating from the Tang Dynasty, has been widely applied in T2DM treatment for thousands of years (Zhang et al., 2014). Despite its clinical hypoglycemic effect, the underlying mechanism is unclear. Recently, an animal study found that HLJDD ameliorated blood glucose and restored the dysregulated microbiota composition. HLJDD-induced hyperglycemia improvement was mainly related to an increase in the abundance of SCFA-producing bacteria, such as *Adlercreutzia, Porphyromonadaceae* (including *Parabacteroides*), *Lachnospiraceae* (including *Blautia*), and a decrease of conditioned pathogenic bacteria, such as *Corynebacteriaceae* (including *Corynebacterium*), *Staphylococcaceae* (including *Staphylococcus*), and *Aerococcaceae* (including *Aerococcus* and *Facklamia*) (Chen et al., 2018).

Besides animal experiments, gradually accumulating clinical studies exploring and verifying the intestinal microbial mechanisms behind the hypoglycemic effect of HM in T2DM treatment have also emerged. They have mainly focused on the modulation of gut microbiota composition. A clinical trial on the anti-diabetic effects of a traditional Chinese herbal formula named Gegen Qinlian decoction (GQD) found a correlation between the increase in the abundance of beneficial gut microbiota and its clinical efficacy, including blood glucose levels and islet β-cell function. Furthermore, the butyrate-producing bacteria *Faecalibacterium prausnitzii* were significantly enriched in the feces of T2DM patients after 12 weeks of intervention (Xu et al., 2015). A randomized clinical trial on the efficacy of a specially designed Chinese herbal formula named AMC for T2DM with hyperlipidemia indicated that AMC significantly ameliorated blood glucose and lipid levels and improved HOMA-IR and triglyceride levels with higher efficacy than metformin. The study confirmed that these effects were associated with butyrate-producing bacteria, such as *Faecalibacterium* spp. and *Blautia* (Tong et al., 2018) (Table 1).

**Table 1:** HM regulates the composition of gut microbiota.

**HM**	**Extract or natural herbs**	**Subject**	**Indexes for therapeutic effects**	**Enriched gut microbiota**	**Reduced gut microbiota**	**References**
Rhubarb	Purified extract (anthraquinone-glycoside)	Type 2 diabetic rats	FBG, GSP, insulin level and sensitivity, GLP-1	*Lactobacillus*	*Lachnospiraceae NK4A136 group, Desulfovibrio*	Cui et al., 2019
Caulis Spatholobi	Water extract	Diet-introduced obesity mice	Body weight, fat organ weight, body fat rate, oxygen consumption, norepinephrine concentration, glucose tolerance, insulin sensitivity, lipids profile, TNF-α, IL-6, IL-1β	*Parabacteroides, Bacteroidetes, Anaerotruncus, Bifidobacterium*	–^b^	Zhang C. H. et al., 2019
Xiexin Tang	Natural herbs^a^	Type 2 diabetic rats	Body weight, FBG, lipids profile, insulin level and sensitivity, TNF-α, IL-6, CRP, resistin	*Adlercreutzia, Alloprevotella, Barnesiella*, [*Eubacterium*] *Ventriosum group, Blautia, Lachnospiraceae UCG-001, Papillibacter, Prevotellaceae NK3B31 group*	–	Wei et al., 2018
Huang Lian Jie Du Decoction	Natural herbs	Type 2 diabetic rats	Body weight, FBG, glucose tolerance, lipids profile, insulin level and sensitivity, IL-1β, IL-6, CRP, MDA, SOD, GSH-Px, ALT, AST, TBA, TBIL, DBIL	*Adlercreutzia, Porphyromonadaceae, Lachnospiraceae*	*Corynebacteriaceae, Staphylococcaceae, Aerococcaceae*	Chen et al., 2018
Gegen Qinlian Decoction	Natural herbs	Patients with T2DM	HbA1c, FBG, 2h-PBG, insulin level and sensitivity, BMI, lipids profile, waist and hip circumferences	*Faecalibacterium prausnitzii*	–	Xu et al., 2015
AMC	Natural herbs	Patients with T2DM	HbA1c, FBG, 2h-PBG, insulin level and sensitivity, lipids profile, BMI, body weight, waist and hip circumferences	*Faecalibacterium* spp, *Blautia*	–	Tong et al., 2018

a *Natural herbs are in a form of formulation, which contains various herbs*.

b *Undifined*.

### HMs Reduce Inflammation Caused by Gut Microbiota and Host Immunity

The pathogenesis of T2DM is closely associated with a low-grade inflammatory state and an activation of the host immune response, also called an ongoing cytokine-induced acute-phase response (Pickup, 2004; Donath and Shoelson, 2011). In T2DM pathogenesis, LPS released by gram-negative bacteria enters the enterohepatic circulation due to an intestinal microecological disorder, which can initiate the immune response in adipose tissue. The expression of TLRs is activated and proinflammatory adipocytokines, such as IL-1, IL-6, and TNF-α are released, resulting in a low-grade inflammatory state (Creely et al., 2007). T cells, as a critical effector of cell-mediated immunity, are crucial for the development of T2DM and the associated inflammation. T cell metabolism is closely related to insulin and its downstream signaling through the insulin receptor, and the lack of insulin receptors on T cells can inhibit glycolysis (Tsai et al., 2018). Besides, accumulating evidence links CD4^+^ T cells to obesity and insulin resistance, which are major risk factors for T2DM (Xia et al., 2017). Subtypes of CD4^+^ T cells, including Th1 and Th2 cells, can produce large amounts of proinflammatory cytokines after their activation to regulate inflammatory processes (Kahn et al., 2006; Raphael et al., 2015). With the accumulating evidence of gut microbiota playing a significant role in host immunity and subsequent inflammation, the related mechanisms of HMs in T2DM treatment are gradually emerging. The therapeutic mechanisms underlying their efficacy have been associated with intestinal anti-inflammation and immunomodulation.

Scutellaria Radix (SR) and Coptidis Rhizome (CR) are well-known herbs applied for diabetes treatment since thousands of years and have demonstrated hypoglycemic effects in both clinical trials and basic experiments (Zhang et al., 2018; Ran et al., 2019). The combination of SR and CR (SC) exerted anti-diabetic activities through the TLR4 signaling pathway involved in anti-inflammation and gut microbiota regulation. The administration of SC in a T2DM KK-Ay mouse model was associated with significantly decreased LPS, IL-6, TNF-α, TLR4, and MyD88 protein levels and improved blood glucose, insulin, and blood lipid content; moreover, *Lactobacillus intestinalis* was considered a possible targeted probiotic (Zhang C. H. et al., 2019).

*Potentilla discolor* Bunge (PDB) is a perennial herb usually utilized as an anti-diabetic agent. In an animal study, its hypoglycemic effect was closely related with the regulation of intestinal endotoxemia and inflammation. Compared with control mice, T2D mice treated with PDB presented a significant decrease in TNF-α, IL-1β, and IL-6 serum levels and LPS in feces and serum. Furthermore, the abundance of the *Bacteroidales_S24-7_group* was increased after PDB administration, accompanied by a decreased abundance of *Helicobacteraceae* (Han et al., 2019).

*Dendrobium* is a HM clinically used for the treatment of T2DM and its complications, and dendrobium polyphenols are its main constituents that exert diverse pharmacological effects (Paudel et al., 2018). A study on the effects of a polyphenol-rich extract of *Dendrobium loddigesii* (DJP) in diabetic mice identified anti-inflammatory activities of DJP for T2DM treatment through a reduction of IL-6 and TNF-α expression, which was correlated with the modulation of gut microbiota, including an increase of the *Bacteroidetes* to *Firmicutes* ratio and *Prevotella/Akkermansia* abundance and a decrease in the abundance of *S24-7*/*Rikenella*/*Escherichia coli* (Li et al., 2018) (Table 1).

### Gut Microbiota-Mediated Metabolism Modulates the Biotransformation of HMs

The clinical application of HMs for T2DM treatment has been widely accepted globally. However, there is still a huge gap in terms of an approval by the US Food and Drug Administration and drug discovery due to the low bioavailability and bioactivity of HMs after oral administration (Kesarwani et al., 2013). In recent years, scientists have found that gut microbiota critically modulate the biotransformation of hypoglycemic HMs, transforming polar and poor lipophilic compounds to less polar and more lipophilic compounds and improving their oral absorption rate for T2DM treatment. Specifically, gut microbiomes encode various enzymes, which can metabolize HMs, modify the structure of the original chemicals, and produce new compounds (Koppel et al., 2017). After the metabolism of gut microbiomes, the bioavailability and bioactivity of newly produced chemical compounds differ from those of the original HM chemicals, and compounds with higher bioavailability and bioactivity can be easily absorbed by the intestine and produce therapeutic effects in the host. During the process of HM biotransformation, a gut bacterial strain can transform various chemical compounds. Meanwhile, a compound can also be transformed by the synergistic effects of various bacteria.

Berberine (BBR) is a representative constituent of a commonly used hypoglycemic HM CR (Wang J. et al., 2019). Numerous studies have demonstrated the hypoglycemic effects of BBR in T2DM (Zhang et al., 2010; Liang et al., 2019). However, as an isoquinoline alkaloid with poor water solubility, BBR is poorly absorbed by intestinal epithelial cells, which leads to its extremely low bioavailability (Liu et al., 2016). Recent studies have found that gut microbiota play an important role in BBR biotransformation and its antidiabetic effects. In the host intestine, gut microbiota convert BBR to a more easily absorbable but inactive metabolite, dihydroberberine (dhBBR), through catalysis of nitroreductases. After dhBBR absorption into intestinal wall tissue, it is oxidized immediately to BBR and exerts pharmacological activities in blood circulation (Feng et al., 2015).

Ginseng is a HM, which has been applied for T2DM treatment for thousands of years. Ginseng beneficial effects in clinical trials include improved blood glucose control and insulin sensitivity in T2DM patients (Gui et al., 2016). Ginsenosides, the main active components of ginseng, exert hypoglycemic effects in type 2 diabetic rats, including lowering blood glucose levels, modulating insulin response, and decreasing body weight (Tian et al., 2018). The metabolism of gut microbiota has a marked influence on ginsenoside pharmacological effects. Ginsenoside Rb1, an anti-diabetic agent belonging to tetracyclic triterpenoid saponins, has low bioavailability (Yu et al., 2018; Zhou P. et al., 2019). However, after catalysis of β-glucosidases produced by gut bacteria, ginsenoside Rb1 can be metabolized to more bioactive compounds, such as ginsenoside 20(S)-Rg3 and compound K (Jung et al., 2012; Quan et al., 2012; Kim, 2013). Ginsenosides Re and Rg1 are also transformed to new metabolites like the rare ginsenosides Rd, GypXVII, Rg2, and protopanaxatriol, which exhibit higher biological and pharmacological activities (Yu et al., 2017).

Curcumin, one of the primary active constituents of the HM turmeric, possesses a range of pharmacological activities including anti-diabetic, anti-inflammatory, and antioxidant effects and is protective against diabetes and its complications (Nabavi et al., 2015; Parsamanesh et al., 2018). However, as a polyphenolic compound, curcumin's poor oral bioavailability represents a big barrier to its clinical efficacy (Lopresti, 2018). Gut microbiota have been identified as a key point in the biotransformation of curcumin. Curcumin can be metabolized through the metabolism of human intestinal bacterium *Blautia sp*. MRG-PMF1 is converted to demethylcurcumin and bisdemethylcurcumin by a methyl aryl ether cleavage reaction (Burapan et al., 2017). Besides, in an *in vitro* human fecal incubation experiment three metabolites, tetrahydrocurcumin (THC), dihydroferulic acid (DFA), and a metabolite tentatively identified as 1-(4-hydroxy-3-methoxyphenyl)-2-propanol, were detected in the mixture containing curcumin after human fecal fermentation (Tan et al., 2015). The bacterium *Escherichia coli* from human feces was discovered in the microbial biotransformation of curcumin. Through a two-step reduction by an unique enzyme purified from *Escherichia coli*, which was named “NADPH-dependent curcumin/dihydrocurcumin reductase”, curcumin could be converted into dihydrocurcumin as an intermediate metabolite and tetrahydrocurcumin as the end product (Hassaninasab et al., 2011).

Quercitrin is a bioflavonoid present in various anti-diabetic HMs, such as Mori Folium, Bupleurum Radix, and hawthorn that has a positive effect on carbohydrate metabolism and antioxidant activities during diabetic treatment (Babujanarthanam et al., 2010, 2011). However, due to its relatively poor bioavailability, the key factors mediating its beneficial effects and underlying mechanism remain elusive. An *in vivo* experiment incubating quercetin with human gut bacteria found that metabolites with higher bioactivity were produced, including quercetin, 3,4-dihydroxyphenylacetic acid, and 4-hydroxybenzoic acid. *Fusobacterium K-60* was detected as the main bacterium, which transformed quercitrin to quercetin (Kim et al., 1999).

## Discussion and Perspectives

In recent years, the mechanisms of T2DM-associated alterations in gut microbiota are being constantly explored. Intestinal dysbiosis, endotoxin-induced metabolic inflammation, immune response disorder, bacterial components and metabolites, and a decreasing production of SCFAs are closely associated with the pathogenesis of T2DM and represent a mechanism of gut microbiota-host interplay. However, research on the hypoglycemic effects of HMs targeting gut microbiota is only in its infancy. Our analysis of relevant clinical studies showed that most merely analyzed the modulation of gut microbiota composition by HMs, whereas the underlying mechanisms, including the interaction mode of floras with HMs, the affected host pathways, and the therapeutic targets were rarely studied. Therefore, we believe that from the perspective of gut microbiota, investigating the therapeutic mechanisms of hypoglycemic HMs has great potential.

HM is the major component of the traditional Chinese medicine (TCM) system and is considered the “basis of all other medicines” (An et al., 2019). For thousands of years, it has not been scientifically recognized, because this system and its theory are totally different from modern science. The core of the TCM system is the balance and imbalance of Yin and Yang, which is difficult to quantify and characterize, whereas modern science focuses on objective and quantifiable evidence. Although prescriptions of multiple HM combinations based on the TCM theory have achieved notable clinical efficacy, the poor knowledge of their mechanisms has greatly limited TCM and HM progress. However, research on the interaction between gut microbiota and HM presents scientific evidence for TCM and HM utility and rational compatibility, which greatly increases the optimism for its use in the clinical practice. In TCM prescriptions, multiple HM combinations exert synergistic or antagonistic effects to obtain better efficacy or reduce toxicity and adverse effects. For example, for the treatment of T2DM, CR, and SR are commonly combined to lower blood glucose, because according to the TCM theory, they both have a bitter flavor and can combat high sugar. As obscure as the theory is, gut microbiota can be a perfect explanation why the main components of CR and SR, namely berberine and baicalin, can improve the imbalance of gut microbiome in the host, increase the abundance of various SCFA-producing bacteria, and decrease the abundance of harmful bacteria (Zhang et al., 2015; Ju et al., 2019). However, an antagonism between CR and SR in the intestine also exists. In an *in vitro* experiment using rat fecal suspensions, CR decreased the bioavailability of anthraquinones by inhibiting the transformation of conjugated anthraquinones to free anthraquinones mediated by gut microbiota, whereas SR confronted CR by inhibiting the glucuronidation of anthraquinones in the intestine (Yan et al., 2015).

The exploration of the interplay of gut microbiota-host-HM also offers new insights into precision HM therapy and drug discovery. Metformin is universally acknowledged as a first-line anti-diabetic agent for the management of T2DM and improves the intestinal bacterial dysbiosis. It has been confirmed that one of the therapeutic effects of metformin in T2DM is through SCFA production as well as a increase of *Eschericha* abundance (Forslund et al., 2015). Metformin exert anti-hyperglycemic effect through an increase in the bile acid gycoursodeoxycholic acid in the intestine by decreasing the abundance of species of *B. fragilis* and its bile salt hydrolase activity (Sun et al., 2018). However, it is less well-known that the origins of metformin can be traced back to a traditional European HM, namely *Galega officinalis* (also known as goat's rue) (Bailey, 2017). This classic and successful example demonstrates that herbs are very valuable for medicine and contribute significantly to drug discovery. With the recent increasing interest and accumulating research evidence on HMs and their pharmacologic effects and related mechanisms, new drug discovery and development based on HMs are expected to be an important future trend. As a definite target, gut microbiota may be a direction for precisely targeted HM therapy and new drug discovery using extracts from natural HMs.

Furthermore, many herbs in the Chinese Pharmacopeia enriched in fibers and botanicals are also present in our daily diet and are named herbal food supplements (Di Giorgi Gerevini et al., 2005). For example, *Momordica charantia* and Chinese yam are common foods in the daily diet and are also used as HMs to treat diabetes and modulate gut microbiota (Li et al., 2017; Wang et al., 2017). Diet is intimately associated with T2DM, and gut microbiota is considered an intersection of diet and disease (Sonnenburg and Backhed, 2016). Furthermore, numerous studies have confirmed that the daily structure is important for the management of diabetes (Nie et al., 2019). Therefore, the exploration of herbal foods regulating the flora may aid the development of hypoglycemic dietary supplements and medications to reach the objective of “Let food be the medicine and medicine be the food.”

## Author Contributions

XT and ML provided the idea. YZ, XG, JT, and LZ wrote the draft of the manuscript. LZ, HG, and YW searched the data and helped with the figures. XY and BP analyzed the data. All authors contributed to the article and approved the submitted version.

## Conflict of Interest

The authors declare that the research was conducted in the absence of any commercial or financial relationships that could be construed as a potential conflict of interest.
